# Changing self-identification among immigrants in the United States

**DOI:** 10.3389/fsoc.2025.1445287

**Published:** 2025-05-09

**Authors:** Ina Chen, Jui-Chung Allen Li

**Affiliations:** ^1^Taipei American School, Taipei, Taiwan; ^2^Quanthon Corporation, Taipei, Taiwan

**Keywords:** immigration, race and ethnicity, self-identity, multi-racial, American Community Survey, racial fluidity

## Abstract

Race and ethnicity are fluid self-identities in the United States, particularly among immigrants, who often redefine their racial and ethnic self-identification as they navigate assimilation and cultural integration. This study uses repeated cross-sectional data from the 2000–2021 American Community Surveys to examine the specific racial and ethnic groups among U.S. immigrants that experienced substantial increases in self-identification. Given that fixed immigration cohorts typically decline in size over time due to emigration and mortality, any observed increase within a cohort indicates individuals reclassifying their reported identity. By controlling for the year of entry into the United States, this analysis employs ordinary least squares (OLS) regressions to estimate annual changes in size and percentage across 46 racial and ethnic categories. The analysis reveals significant increases in identification with multiracial whites and single-race or multiracial “Write-In” groups—categories not printed in the survey questionnaire. These findings underscore the fluidity and complexity of ethnic identities and highlight a shift from broad racial classifications to more specific identities that reflect heritage more accurately. These insights contribute to a broader understanding of identity dynamics and a growing diversity and inclusivity within the U.S. racial and ethnic landscape.

## Introduction

In the United States, the concept of race has traditionally been considered a fixed category defined by ancestry and regarded as biologically inherent (Fredrickson, 2002; Davenport, 2020). However, the interpretation and definition of racial and ethnic groups vary over time and context, shaped by the evolving socio-political landscape of contemporary American society (Barth, 1969; Haney López, 1996; Cornell and Hartmann, 1998). Thus, many have argued that the choice of racial and ethnic identity should be fluid and a matter of personal choice (Goyette and Xie, 1999). People often reassess and modify their racial and ethnic identity to best describe them, though these changes rarely happen spontaneously and are instead driven by contextual influences (Liebler et al., 2017).

With 45 million immigrants (or one in seven) of the population, the United States serves as an ideal setting for studying immigrants’ racial and ethnic identity (U.S. Economy and the Federal Budget, 2023). Compared to the native-born population, immigrants tend to exhibit a more fluid sense of identity as they assimilate and intermarry, contributing to an especially nuanced projection of America’s racial and ethnic diversity (Edmonston and Passel, 1994; Edmonston et al., 2002; Gibson, 1992; Perez and Hirschman, 2009). While describing their country of origin might not pose an issue, immigrants frequently struggle to navigate the American classification of race and ethnicity. Many new arrivals lack an understanding of the established categories, and their level of assimilation further influences how they perceive and adopt their unique American identity (Doyle and Kao, 2007; Lee and Bean, 2004; Harris and Sim, 2002). Factors such as countries of origin, social classes, geographic location, and cultural practices create varying degrees of integration (Waters, 1990; Alba and Nee, 2003). Thus, the racial and ethnic identities of immigrants may be particularly prone to change over time.

Nonetheless, few studies have documented the specific groups exhibiting systematic changes within their racial and ethnic identities. This present paper seeks to fill in this gap and explore which immigrant groups have observed the highest number or percent change in racial and ethnic identities.

### Prior studies on the fluidity of racial and ethnic identity

Prior research recognizes the fluidity of self-reported racial and ethnic identity. Liebler et al. (2017) examined patterns and directions of individual-level response change across all federally defined racial and ethnic groups in the United States, shedding light on the countervailing flows of individuals entering and exiting each category. Situational and contextual factors also impact how respondents perceive and report their identity. Harris and Sim (2002) revealed that 12% of adolescents report different racial identities at home and school, suggesting that identity can be relational to social environments. Similarly, Saperstein and Penner (2012), using data from the National Longitudinal Survey of Youth 1979, asserted that racial classification is a flexible propensity for both self-identification and interview classification. However, there have been debates regarding this analysis by Kramer et al. (2016) and Alba et al. (2016) on how racial fluidity is less common than Saperstein and Penner asserted. They raised questions on interviewer effects and biases, such as being less exposed to ethnoracial diversity, and measurement issues, such as not including weights, which led to failure in properly adjusting for over-and undersampling. Despite these disagreements, our paper is distinct because it does not exhibit these issues and, furthermore, adds nuances to the broader study of racial fluidity and shifts in self-identification.

With racial identity already fluid in the United States, this variability is even more pronounced among immigrants as they adapt to life in a new country. Mowen and Stansfield (2015), using a longitudinal structural equation model, found that children of immigrants with high self-esteem, self-worth, and strong family cohesion are more likely to change their racial identification over time, while socioeconomic status and depression have little effect. Waters (2004) argued that employment and social class shape the development of racial and ethnic identities among immigrants. Among second-generation immigrants, those from middle-class backgrounds are more likely than others to embrace the American identity by positioning themselves as members of the “model minority.” Similarly, middle-class immigrants tend to experience greater structural assimilation, whereas working-class immigrants with lower educational levels lack the social interactions necessary to reshape their racial and ethnic self-perceptions (Roth, 2012). These studies underscore the role of socioeconomic factors in assimilation and identity formation, yet they do not examine the specific immigrant groups that are more likely to alter their racial and ethnic identities over time (DeFina and Hannon, 2016).

The highly fluid nature of race for mixed-race individuals is a relatively recent development (Davenport, 2020). Before the 2020 Census, Americans could not self-identify with more than one race. Liebler (2016) found that in 1960, most children born to Asian-white and Black-white parents identified under the minority race of their parents. By 2010, the majority of such individuals identified as multiracial. The increase in multiracial and mixed-ancestry Asian responses will be discussed in our paper. Similarly, in 1960 and 1970, many half-white respondents only reported as white, but after 1970, there was a major shift away from monoracial white responses as “multiracial” became a more widely used option (Liebler, 2016). The growing recognition and acceptance of mixed-race identities indicate a broader societal trend toward more flexible and inclusive racial boundaries.

### Methodological issues in documenting changes in racial and ethnic identities

Tracking changes in self-reported racial and ethnic identities at the individual level over an extended period presents a significant challenge, as direct longitudinal measurements of the same individual’s racial and ethnic identities are often unavailable. We thus propose an indirect approach using the rationale and insights first outlined in Coale (1955). Specifically, Coale compared the “observed” pattern of population distributions with the “expected” pattern, attributing any discrepancies to misclassification or other measurement errors in the census headcounts. This analysis assumes that closed populations have zero net migration and no unusual mortality or fertility rates (p. 17–20). Specifically, Coale argued that age-specific sex ratios ought not suddenly change between adjacent age groups. Similarly, cohort-specific age ratios ought not to suddenly change between adjacent cohorts. Hence, the “expected” age patterns of sex ratios and cohort patterns of age ratios ought to be smooth. The “observed” abrupt edges, thus, would indicate errors.

Using a simplified variant of this method, Perez and Hirschman (2009) analyzed U.S. Census and American Community Survey data from 1980 to 2006, concluding that the observed population growth of major race and ethnic subpopulations could not be fully explained by natural increase and immigration. They suggested that changes in identity preference have contributed to this observed increase. Liebler and Ortyl (2014) applied a similar approach to predict the population size of American Indians in 2000 by comparing their estimate with the actual Census count. Their findings revealed that actual counts far surpassed projections across all age groups, indicating a growing tendency for individuals who had not previously identified as American Indian to do so. Expanding this framework to historical and geopolitical contexts, Chen and Li (2023) analyzed how Taiwan’s political relationships with China and the United States influence ethnic identification among Taiwanese. Examining fixed-immigration cohorts using data from the 2001 to 2019 American Community Survey, they documented an increasing number of individuals identifying as Taiwanese, accompanied by a corresponding decline in those identifying as Chinese or reporting only Taiwanese ancestry. This pattern highlighted the significant impact of geopolitical relationships on identity formation among immigrants and aligned with Caron et al. (2023), who argued that identity reclassification occurs as immigrants assimilate and reshape their perception of national identity. While both Perez and Hirschman (2009) and Liebler and Ortyl (2014) tracked changes in self-reported racial and ethnic identities, neither studied identity shifts among immigrants. Similarly, Liebler and Ortyl (2014) and Chen and Li (2023) exclusively focused on single racial and ethnic groups. This present paper will examine the full spectrum of racial and ethnic groups of immigrants, thereby improving this line of scientific inquiry.

## Methods

### The rationale for identifying changing racial and ethnic identities

Our rationale for identifying changes in racial and ethnic identity follows Coale (1955) framework. We compare, for each racial and ethnic group of immigrants, the “observed” period trends in the size of immigration cohorts with the “expected” period trends in the size of immigration cohorts. Imagine the immigrants from racial and ethnic group *j* who moved to the United States in year *k*. The size of such a cohort of immigrants, denoted by $y_{jk}$ , ought to *only* decrease over time through emigration and deaths—because, by definition and construction, one can be born into a birth cohort but not an *immigration* cohort. Anyone who immigrated in an earlier (or a later) year will count as part of an earlier (or a subsequent) immigration cohort. We will, in theory, attribute any positive deviation from the “expected” decline as people change identities into that racial and ethnic category.

Using American Community Survey (ACS) data collected in year *t*, we estimate the size of an immigration cohort, $y_{jkt}$ , for racial and ethnic group *j* that arrived in the United States in year *k*. We use ordinary least squares (OLS) regressions with survey year *t* as the only predictor to see how the size of race/ethnicity-specific immigration cohort *jk* changes over time *t.* We conduct a parallel set of OLS regression models using the natural logarithm of cohort size, $\ln\left(y_{jkt}\right)$ , as the dependent variable to approximate the percent change in cohort size.

A major challenge in Coale’s identification approach is that the slope of the “expected” period decline for each racial and ethnic group is unknown because the race−/ethnicity-specific emigration and mortality rates for a given period are unknown. Even more challenging is that such race−/ethnicity-specific emigration and mortality rates might not be knowable if people change their reported race and ethnicity. Thus, we adopt a relatively conservative strategy by assuming zero slope as the “expected” period trend. Hypothetically, a more precise baseline for distinguishing between natural population decreases and increases would be a number below zero. In other words, we will only interpret that people change their identity into a racial and ethnic category if we identify a positive period trend (not just a positive deviation from a potential decline) in the size of an immigration cohort. As our analysis is mainly a methodological exercise, our interpretations tend to be inductive, rather than deductive, in the spirit of exploratory data analysis (Tukey, 1977).

While our methodological approach may appear to resemble the pseudo-cohort method, the two are different. Both construct cohorts of respondents from repeated cross-sectional surveys, but the pseudo-cohort approach examines how age-related attributes change over time, whereas our approach focuses exclusively on cohort size. While the pseudo-cohort approach is closely related to panel data analysis in econometrics (see, e.g., Deaton, 1985) and its applications (Li and Tsui, 2016), our approach is akin to Coale’s (1955) and Perez and Hirschman’s (2009) demographic analysis of census and ACS data. More specifically, unlike studies that analyze individual-level/non-aggregate attributes (e.g., health, education, and work of the respondents as in Li and Tsui, 2016), our approach uses cohort-level/aggregate attributes and does not involve any individual-level attributes. Relatedly, our study refers to the entire population at multiple snapshots rather than a probability sample. Hence, we do not use multilevel analysis, which adjusts for standard errors due to non-independence between observations and accounts for cross-level interaction effects, in this context for this paper.

### Data and variables

We use the 2000–2021 ACS data provided by the Integrated Public Use Microdata Series (Ruggles et al., 2022). The ACS is a large-scale, multiyear, repeated cross-sectional survey designed to replace the Census Long Form and gather information on the socioeconomic and demographic characteristics of individuals in residential households. It provides snapshot measurements of the U.S. population not living in group quarters and is representative of the U.S. population. The ACS employs a stratified random sampling method by selecting addresses from the Master Address File, a comprehensive database of all known residential addresses in the United States, Puerto Rico, and associated island areas maintained by the U.S. Census Bureau. Annually, approximately 3.5 million addresses are surveyed through mailed questionnaires, telephone interviews, and in-person visits. The survey is conducted monthly, capturing a dynamic, time-sensitive picture of the country over time. The data were weighted to better align the characteristics of the sample with those of the full U.S. population (U.S. Census Bureau, 2022).

Our primary variable of interest is self-identified race and ethnicity, for which we extracted detailed race and ethnicity information from the IPUMS-constructed variable RACED. The ACS employed a consistent race questionnaire design based on self-identification from 2000 to 2021. The racial and ethnic categories “generally reflect a social definition of race recognized in [the United States] and [are] not an attempt to define race biologically, anthropologically, or genetically. It is recognized that the categories of the race item include racial and national origin or sociocultural groups” (U.S. Census Bureau, 2018). Since detailed RACE categories encompass a broad spectrum of immigrants, we can perform a comprehensive analysis of the diverse immigrant population residing in the country.

We created immigration cohorts using the IPUMS-constructed variable, YRIMMIG, which indicates the year a foreign-born individual entered the United States, or the most recent year in instances of multiple entries. We extracted the years 1960 to 1999 and subdivided this period into four decadal immigration cohorts using dummy variables. Considering that 48% of all U.S. immigrants arrived before 2000, this timeframe offers a robust representation of the immigrant composition in the United States (Ward and Batalova, 2022).

Recall bias could impact the accuracy of ACS measurements for a respondent’s year of first entry into the United States. Some individuals may forget their exact year of arrival or confuse multiple entries, leading to a possible source of error. The YRIMMIG question may also be interpreted differently than intended, and respondents might have complex immigration histories that could not be neatly defined in a single year. Despite these potential sources of error, even with recall bias present, we have no reason to suspect that they would disproportionately affect any specific racial and ethnic group.

In our sample, we included only non-native-born respondents with valid data on their first entry into the United States and the IPUMS-constructed RACED variable. To ensure the reliability of our estimated size of each immigration-cohort-by-survey-year cell and the overall estimated trends by survey year, we filtered cases based on three criteria: (1) exclusion of respondents listed under “four or more major race groups” (i.e., keeping only those with three or fewer major race groups); (2) exclusion of races and ethnicities with less than 6 years of survey data available between 2000 and 2021 as they lack sufficient data for regression analysis; and (3) exclusion of races and ethnicities where the majority of immigration cohort observations per survey year included fewer than 30 respondents to maintain statistical reliability. The original ACS dataset contained 252 RACED categories. Following our selection procedure, we identified 46 race and ethnicity categories for analysis.

To assess the robustness of our results, we conducted a sensitivity analysis by refining the categorization of immigration cohorts and race categories. Using the same immigration years (1960 to 1999), we subdivided the original four decadal immigration cohorts into 8 five-year cohorts for greater granularity. Additionally, we consolidated the 46 individual race and ethnic categories into seven broader categories: White, Black/African American, Asian, Two or More Asian Races, Pacific Islanders, Other Race, and Two Major Race Groups. The sensitivity analysis resulted in six additional tables, incorporating two analytical approaches (headcount and natural logarithm), two immigration cohort classifications (decadal and five-year), and two racial classifications (individual and grouped). The primary results are presented in two main tables, with the remaining six tables for the sensitivity analysis included in Supplementary Appendix.

We do not think it is necessary or appropriate to report the standard errors because, while our data structure may look like a multilevel model, our outcome measures are headcounts and logged headcounts estimated from the ACS raw data. Moreover, there are only 22 data points in each regression. Testing the significance of the coefficients against sampling variability is not informative.

Table 1 displays basic demographic characteristics for the 46 individual race and ethnicity categories. Most groups show relatively balanced gender distributions, with a handful of outliers: the Japanese included 67.9% females, while Nepalese reported only 41.9% females. The average age ranges from 36 to 52, and the average educational attainment generally clusters between 12 and 14 years of schooling, with some categories as low as 9 and Fijians as high as 15. Still, any dispersion or variations would not affect the robustness of our analysis, but they may be the potential reasons or factors influencing self-identification trends. However, investigating specific, individual case analyses for reasons of change will be a task for future research.

**Table 1:** The basic demographic of descriptive statistics of race categories.

Race categories	% female	Mean age (SD)	Mean years of schooling (SD)
100 White	50.0%	45.9 (16.87)	11.69 (4.58)
200 Black/African American	52.5%	45.99 (16.31)	12.97 (3.72)
400 Chinese	53.8%	49.78 (16.74)	13.48 (4.95)
410 Taiwanese	53.8%	52.14 (16.04)	15.87 (3.41)
500 Japanese	67.9%	51.2 (17.39)	14.18 (3.32)
600 Filipino	58.8%	50.55 (16.47)	14.12 (3.21)
610 Asian Indian	46.7%	46.18 (15.92)	14.9 (3.94)
620 Korean	58.3%	47.68 (17.33)	13.92 (3.72)
640 Vietnam	50.4%	47.49 (16.07)	12.08 (4.67)
643 Nepalese	41.9%	45.63 (13.16)	14.24 (4.74)
660 Cambodian	53.4%	46.15 (15.67)	10.19 (5.66)
661 Hmong	50.3%	40.76 (15.96)	9.68 (6.15)
662 Laotian	49.9%	45.52 (14.77)	10.5 (5.31)
663 Thai	63.5%	50.23 (14.87)	13.08 (4.39)
664 Bangladeshi	42.9%	43.79 (14.9)	13.78 (4.17)
665 Burmese	49.8%	51.46 (14.82)	13.24 (5.18)
666 Indonesian	52.7%	45.37 (15.83)	14.42 (3.15)
669 Pakistani	42.7%	44.87 (15.75)	14.11 (4.02)
670 Sri Lankan	48.1%	48.63 (15.96)	14.96 (3.37)
671 All other Asian	50.7%	48.65 (15.73)	13.54 (4.69)
672 Asian, not specified	49.1%	39.15 (15.62)	12.63 (5.02)
674 Chinese & Filipino	59.6%	47.18 (15.97)	14.96 (2.8)
675 Chinese & Vietnamese	51.0%	48.07 (14.69)	11.62 (5.19)
676 Chinese and Asian Write-in; Chinese and Other Asian	55.3%	49.03 (15.18)	13.0 (4.98)
678 Asian Indian & Asian Write-in	43.6%	48.54 (15.66)	13.4 (4.87)
680 Saman	50.7%	45.35 (15.71)	12.27 (3.31)
685 Chamorro	51.3%	42.46 (16.17)	12.12 (3.72)
695 Fijian	51.4%	51.32 (13.68)	12.31 (3.85)
697 One or more Melanesian races (2000, ACS)	51.5%	39.75 (15.3)	12.11 (3.7)
699 Pacific Islander	55.4%	44.6 (15.57)	12.59 (3.68)
700 Other Race, N.E.C.	47.8%	42.66 (15.12)	9.79 (4.51)
801 White and Black	52.3%	42.05 (16.62)	12.24 (4.38)
802 White and AIAN	45.9%	42.97 (15.19)	11.39 (4.61)
811 White and Chinese	55.0%	40.82 (17.41)	13.36 (4.47)
812 White and Japanese	52.2%	40.42 (15.62)	13.88 (3.61)
813 White and Filipino	57.6%	37.37 (16.37)	13.39 (3.43)
815 White and Korean	54.7%	36.41 (14.66)	13.6 (3.51)
817 White and Asian Write-in	47.6%	44.34 (16.6)	13.2 (4.38)
818 White and other Asian Race	45.4%	50.27 (15.89)	13.79 (4.22)
826 White and “other race” Write-in	50.0%	51.32 (15.78)	10.97 (4.75)
845 Black and “other race” Write-in	53.2%	50.04 (16.8)	12.47 (4.08)
856 AIAN and “other race” Write-in	46.7%	48.32 (15.21)	10.37 (4.97)
865 Filipino and PI Write-in	57.4%	51.33 (15.97)	14.14 (2.89)
867 Asian Write-in and PI Write-in	54.8%	43.86 (15.79)	13.43 (3.47)
885 Asian Write-in and “other race” Write-in	49.7%	49.8 (15.36)	13.29 (4.75)
907 White and American Indian/Alaska Native and “other race” Write-in	49.2%	49.56 (15.23)	11.68 (4.94)

## Results

Table 2 presents the regression coefficients for change in race and ethnicity (RACED variable, constructed by the IPUMS), holding constant immigration cohorts (1970s, 1980s, and 1990s, with 1960s as the reference category). The coefficient of survey year represents the annualized average change in cohort size for each race and ethnicity. A positive coefficient indicates an increase in cohort size between 2000 and 2021. Since cohort sizes can only decrease over time due to mortality and emigration, we interpret any increase as consistent with respondents in a particular race and ethnicity changing their identity from other races and ethnicities into the particular RACED category.

**Table 2:** Regression coefficients of average headcount change by race categories from 2000 to 2021.

		Immigration cohorts					
Race categories	Survey Year	1970–79	1980–89	1990–99	Constant	*R^2^*	*n*
826 White and “Other Race” Write-in	**20,777**	35,220	105,101	164,157	−161,410	0.20	88
817 White and Asian Write-in	**1,388**	4,248	6,155	7,943	−5,152	0.41	48
867 Asian Write-in and PI Write-in	**1,141**	2,320	3,107	3,618	−5,697	0.52	44
818 White and Other Asian Race	**897**	9,208	13,836	17,544	−6,336	0.70	60
845 Black and “Other Race” Write-in	**868**	3,985	9,442	12,418	−4,303	0.22	88
669 Pakistani	**623**	12,739	36,436	69,048	−4,211	0.95	88
671 All Other Asian	**503**	3,316	8,149	12,768	−4,140	0.70	88
856 AIAN and “Other Race” Write-in	**499**	1,468	4,153	6,111	−3,995	0.52	88
801 White and Black	**417**	3,878	16,700	30,723	4,108	0.88	88
907 White and American Indian/Alaska Native and “Other Race” Write-in	**402**	587	1,782	3,150	−3,466	0.24	88
885 Asian Write-in and “Other Race” Write-in	**395**	2,339	5,034	7,041	−3,349	0.54	88
410 Taiwanese	**336**	9,489	22,186	18,463	1,031	0.85	60
664 Bangladeshi	**331**	1,678	8,112	28,062	−3,044	0.94	88
802 White and AIAN	**319**	1,634	5,469	10,799	1,983	0.69	88
865 Filipino and PI Write-in	**254**	3,127	3,255	3,603	−1,822	0.53	60
643 Nepalese	**237**	679	2,452	8,499	−3,642	0.91	40
675 Chinese and Vietnamese	**152**	6,519	11,470	5,729	−1,364	0.82	88
665 Burmese	**140**	1,469	3,437	9,567	−1,842	0.96	88
692 One or More Melanesian Races	**125**	840	3,164	6,680	−420	0.65	48
678 Asian Indian and Asian Write-in	**107**	1,709	3,870	5,416	−795	0.71	88
699 Pacific Islander	**93**	1,276	3,217	5,129	−317	0.52	88
812 White and Japanese	**92**	−1,008	−2,171	−179	5,158	0.39	88
676 Chinese and Asian Write-in; Chinese and Other Asian	**91**	1,475	4,888	3,707	−546	0.70	88
661 Hmong	**62**	15,239	35,349	26,953	−215	0.94	88
815 White and Korean	**50**	3,347	3,023	1,570	2,228	0.59	88
674 Chinese and Filipino	**43**	1,645	3,408	2,748	428	0.69	88
690 Fijian	**30**	1,370	3,634	5,508	−25	0.83	40
670 Sri Lankan	−4	1,943	4,903	7,011	516	0.87	88
813 White and Filipino	−13	3,855	6,201	6,355	3,467	0.71	88
680 Saman	−68	3,847	3,383	4,137	3,683	0.49	88
811 White and Chinese	−70	1,587	1,630	3,361	2,509	0.41	88
666 Indonesian	−105	−488	4,121	13,373	3,810	0.89	88
660 Cambodian	−112	11,579	79,291	16,753	1,971	0.98	88
663 Thai	−140	23,935	17,684	18,803	6,881	0.93	88
685 Chamorro	−275	2,113	4,034	5,277	6,438	0.46	88
662 Laotian	−322	18,723	68,113	16,301	4,053	0.94	88
640 Vietnam	−1,105	159,845	253,480	373,399	19,505	0.99	88
500 Japanese	−1,319	6,614	9,111	38,683	46,471	0.67	88
620 Korean	−2,148	154,869	231,450	198,577	54,454	0.98	88
600 Filipino	−2,478	162,065	301,832	330,672	118,255	0.99	88
672 Asian (not specified)	−2,530	11,372	27,126	35,386	16,993	0.63	48
400 Chinese	−3,645	146,978	394,714	532,475	138,153	0.99	88
610 Asian Indian	−4,055	129,446	285,024	548,982	86,386	0.98	88
200 Black/African American	−6,012	198,791	542,379	745,965	232,704	0.99	88
700 Other Race, N.E.C.	−19,282	436,706	1,170,284	1,843,069	509,566	0.94	88
100 White	−63,163	362,987	1,511,961	3,448,352	2,483,274	0.89	88

Bold survey year values indicate categories with a positive logarithmic regression coefficient for change in race and ethnicity.

Table 2 ranks the 46 RACED categories by the coefficient of the survey year. The greatest annual average increase in immigration cohort size (first row) is 20,777 persons per year for “White and “Other Race” Write-In.” The second largest increase is 1,388 persons per year for “White and Asian Write-In.” The greatest annual average decrease in immigration cohort size (last row) is “White,” which loses 63,163 persons per year. This magnitude of decrease in “White” suggests that this category gradually loses members to “White and “Other Race” Write-In” and “White and Asian Write-In” besides natural decreases in mortality and emigration. Of the 46 RACED categories, 27 exhibit a positive coefficient, which indicates an increase across successive years of the ACS.

The estimates in Table 2 do not account for the difference in the sizes of racial and ethnic groups, such as White being significantly larger than others (e.g., Sri Lankan). Table 3 presents regression coefficients of models predicting the logarithms of the headcounts, which we interpret as the annualized percent change in the size of immigration cohorts.

**Table 3:** Regression coefficients of average logged headcount change by race categories from 2000 to 2021.

		Immigration cohorts					
Race categories	Survey year	1970–79	1980–89	1990–99	Constant	*R^2^*	*n*
867 Asian Write-in and PI Write-in	**0.593**	2.342	2.404	2.473	1.005	0.52	44
692 One or more Melanesian Races	**0.385**	1.845	2.809	4.638	1.970	0.52	48
865 Filipino and PI Write-in	**0.337**	2.633	1.268	1.090	1.322	0.65	60
885 Asian Write-in and “Other Race” Write-in	**0.270**	2.360	3.066	2.958	1.961	0.57	88
907 White and American Indian/Alaska Native and “Other Race” Write-in	**0.221**	0.954	0.698	1.961	2.525	0.39	88
817 White and Asian Write-in	**0.156**	0.773	1.063	1.277	6.723	0.56	48
856 AIAN and “Other Race” Write-in	**0.116**	0.652	1.364	1.539	5.646	0.79	88
664 Bangladeshi	**0.109**	2.249	4.077	5.336	3.752	0.72	88
678 Asian Indian and Asian Write-in	**0.108**	2.285	3.246	3.310	3.747	0.48	88
643 Nepalese	**0.107**	1.367	2.533	3.788	3.492	0.90	40
671 All Other Asian	**0.094**	1.561	2.264	2.825	5.611	0.71	88
818 White and Other Asian Race	**0.093**	1.090	1.266	1.489	7.105	0.79	60
676 Chinese and Asian Write-in; Chinese and Other Asian	**0.069**	1.850	2.633	2.437	4.766	0.37	88
661 Hmong	**0.048**	4.493	5.324	5.046	4.646	0.81	88
675 Chinese and Vietnamese	**0.046**	4.028	4.592	3.848	4.260	0.82	88
699 Pacific Islander	**0.040**	1.046	1.755	2.065	5.955	0.70	88
662 Laotian	**0.038**	4.195	5.452	4.056	5.269	0.81	88
802 White and AIAN	**0.037**	0.253	0.645	1.069	8.157	0.68	88
826 White and “Other Race” Write-in	**0.033**	0.448	1.023	1.276	9.701	0.23	88
665 Burmese	**0.030**	1.539	2.299	3.249	5.459	0.89	88
801 White and Black	**0.027**	0.348	1.117	1.557	8.709	0.82	88
845 Black and “Other Race” Write-in	**0.025**	0.534	1.045	1.181	7.965	0.39	88
670 Sri Lankan	**0.025**	2.169	2.994	3.337	5.301	0.66	88
812 White and Japanese	**0.024**	−0.267	−0.456	−0.038	8.451	0.36	88
669 Pakistani	**0.022**	1.951	2.897	3.503	7.419	0.97	88
690 Fijian	**0.022**	1.404	2.310	2.706	5.602	0.85	40
410 Taiwanese	**0.019**	1.081	1.702	1.535	8.259	0.92	60
660 Cambodian	**0.019**	3.282	5.158	3.614	5.932	0.85	88
674 Chinese and Filipino	**0.015**	1.189	1.754	1.568	6.422	0.67	88
815 White and Korean	**0.015**	0.802	0.738	0.417	7.734	0.55	88
811 White and Chinese	**0.007**	0.946	0.955	1.342	7.045	0.26	88
813 White and Filipino	−0.001	0.748	1.051	1.073	8.100	0.80	88
640 Vietnam	−0.001	3.073	3.516	3.893	8.967	1.00	88
663 Thai	−0.006	1.702	1.461	1.512	8.641	0.96	88
666 Indonesian	−0.010	−0.181	0.941	1.818	7.948	0.88	88
600 Filipino	−0.010	1.017	1.456	1.527	11.533	0.99	88
400 Chinese	−0.012	0.908	1.604	1.851	11.629	1.00	88
680 Saman	−0.012	0.911	0.835	0.896	8.021	0.56	88
610 Asian Indian	−0.012	1.376	2.013	2.604	10.812	1.00	88
620 Korean	−0.012	1.770	2.114	1.976	10.493	0.99	88
200 Black/African American	−0.014	0.783	1.442	1.695	12.173	0.99	88
700 Other Race, N.E.C.	−0.019	0.889	1.579	1.960	12.807	0.97	88
500 Japanese	−0.023	0.197	0.258	0.746	10.619	0.85	88
100 White	−0.024	0.178	0.591	1.051	14.650	0.85	88
685 Chamorro	−0.031	0.495	0.715	0.887	8.419	0.52	88
672 Asian (not specified)	−0.096	1.492	2.204	2.563	8.436	0.87	48

Bold survey year values indicate categories with a positive regression coefficient for change in race and ethnicity.

Table 3 ranks the 46 RACED categories by the percent change per survey year. The greatest annual average increase in immigration cohort size (first row) is 59.3% per year for “Asian Write-In and PI (Pacific Islander) Write-In.” The greatest annual average decrease in immigration cohort size (last row) is “Asian (not specified),” which loses 9.6% per year. This magnitude of decrease in “Asian (not specified)” might suggest that this category is losing members to the “Asian Write-In and PI (Pacific Islander) Write-In.” category in addition to natural decreases in mortality and emigration. Of the 46 RACED categories, 31 exhibit a positive coefficient or an increase across successive years of the ACS.

Table 4 compares the RACED categories with annual increases from Tables 2, 3 for clearer visualization of the relative magnitude between headcount change and proportional percent change. The “White and “Other Race” Write-In” category recorded the largest increase in sheer cohort size, gaining approximately 20,777 persons per year. Yet, this increase translates to a 3% annual growth, making it less significant in proportional terms. The second largest annual average increase in immigration cohort size using the logarithm of the headcounts is “One or More Melanesian Race,” which gains about 38.5% per year. However, this significant increase only corresponds to an additional 125 persons per year because the size of this RACED group is small. The overall patterns of sheer and proportional changes are trending in the same direction, except for four RACED categories. These categories show a positive coefficient in the right panel (percent change) but do not exhibit a corresponding positive coefficient in the left panel (headcount change) of Table 4, suggesting the four pairs of results might not be too robust: Laotian (*b* = −322), Sri Lankan (*b* = −4), Cambodian (*b* = −112), and “White and Chinese” (*b* = −70). These discrepancies between trends in sheer cohort size (measured by headcounts) and trends in percentage, observed in a few categories, should warrant caution against overgeneralization and highlight the need for further research into the underlying causes of these variations. The remaining 27 RACED categories are highly robust, with both measures reflecting positive growth in the size of successive immigration cohorts.

**Table 4:** Positive regression coefficients for headcount and logged headcount of race categories.

Race categories	Count (27/46)	Race categories	Log (31/46)
826 White and “Other Race” Write-In	20,777	867 Asian Write-In and PI Write-In	0.593
817 White and Asian Write-In	1,388	692 One or more Melanesian races (2000, ACS)	0.385
867 Asian Write-In and PI Write-In	1,141	865 Filipino and PI Write-In	0.337
818 White and Other Asian Race	897	885 Asian Write-In and “Other Race” Write-In	0.270
845 Black and “Other Race” Write-In	868	907 White and American Indian/Alaska Native and “Other Race” Write-In	0.221
669 Pakistani	623	817 White and Asian Write-In	0.156
671 All other Asian	503	856 AIAN and “Other Race” Write-In	0.116
856 AIAN and “Other Race” Write-In	499	664 Bangladeshi	0.109
801 White and Black	417	678 Asian Indian and Asian Write-In	0.108
907 White and American Indian/Alaska Native and “Other Race” Write-In	402	643 Nepalese	0.107
885 Asian Write-In and “Other Race” Write-In	395	671 All Other Asian	0.094
410 Taiwanese	336	818 White and other Asian Race	0.093
664 Bangladeshi	331	676 Chinese and Asian Write-In; Chinese and Other Asian	0.069
802 White and AIAN	319	661 Hmong	0.048
865 Filipino and PI Write-In	254	675 Chinese and Vietnamese	0.046
643 Nepalese	237	699 Pacific Islander	0.040
675 Chinese and Vietnamese	152	**662 Laotian**	0.038
665 Burmese	140	802 White and AIAN	0.037
692 One or More Melanesian races	125	826 White and “Other Race” Write-In	0.033
678 Asian Indian and Asian Write-In	107	665 Burmese	0.030
699 Pacific Islander	93	801 White and Black	0.027
812 White and Japanese	92	845 Black and “Other Race” Write-In	0.025
676 Chinese and Asian Write-In; Chinese and Other Asian	91	**670 Sri Lankan**	0.025
661 Hmong	62	812 White and Japanese	0.024
815 White and Korean	50	669 Pakistani	0.022
674 Chinese and Filipino	43	690 Fijian	0.022
690 Fijian	30	410 Taiwanese	0.019
		**660 Cambodian**	0.019
		674 Chinese and Filipino	0.015
		815 White and Korean	0.015
		**811 White and Chinese**	0.007

The four bold race and ethnic categories represent the only cases with a positive percent change coefficient and a negative headcount change coefficient.

### Sensitivity analysis

The sensitivity analysis (see Supplementary Tables 1–6) confirms the robustness of our findings, showing that the observed growth in the size of successive immigration cohorts for many racial and ethnic categories is not a byproduct of our decision to group immigration cohorts or racial and ethnic groups in one way or another. First, whether we group immigration cohorts into 10-year or 5-year categories does not affect the results. In the headcount analysis, all the regression coefficients for the eight (5-year) groups of immigration cohorts are, as expected, exactly half those of the four (10-year) groups of immigration cohort analysis. Since each cohort in the eight-group model is half the size of its corresponding cohort in the four-group model, as explained by the expected relationship between cohort size and magnitude of change, the headcount will also change proportionally. Moreover, in the analysis using the natural logarithm of cohort size, the regression coefficients for the four (10-year) and eight (5-year) cohort models remain similar, differing by less than 0.5% in most cases, thus reinforcing the consistency and robustness of our results.

The broader racial classification results further corroborate our finding that more Americans change their racial and ethnic identities to multiracial categories. Among the seven racial categories, “Two or More Asian Races” and “Two Major Race Groups” stand out as the only categories showing positive regression coefficients, which indicates more people shifting into these categories from 2000 to 2021, across both analytical approaches and immigration cohort classifications.

## Discussion

In this study, we asked, “Which immigrant groups in the United States have experienced a growth in their racial and ethnic identification?” Our analytic approach is built on the insight described in Coale (1955) that once an immigration cohort of a particular racial and ethnic group arrives in the United States, the size of this cohort can only shrink over time due to mortality or emigration. We interpreted any observed increase in the size of an immigrant cohort over time as individuals originally identifying outside that group changing their racial and ethnic identities into that particular group.

Our empirical results revealed significant shifts in racial and ethnic self-identification among immigrants in the United States from 2000 to 2021, as evidenced by the increasing trends in the size of immigration cohorts across different racial and ethnic categories, especially among those identifying as “White and “Other Race” Write-In” and “White and Asian Write-In.” Corresponding to these trends, we observed a parallel move away from singular, fixed racial and ethnic identities, with the “White (only)” category, in particular, experiencing the most substantial annual reduction in size. The result underscores a notable increase in the adoption of mixed-race and ethnic identities, particularly those requiring extra efforts of “write-in.” Such a dynamic nature of racial and ethnic self-identification is further highlighted by the results on the annualized percent changes, indicating substantial growth in categories representing more specific or mixed identities, such as “Asian Write-In and PI (Pacific Islander) Write-In.” These findings pin down the specific racial and ethnic categories most influenced, as described by arguments and previous empirical findings that racial and ethnic identities are fluid among immigrants (Edmonston and Passel, 1994; Edmonston et al., 2002; Gibson, 1992; Perez and Hirschman, 2009).

The observed shifts likely reflect broader social and cultural transformations that influence individuals’ racial and ethnic perceptions and self-identifications. The increase in mixed-race and ethnic identifications confirms the nuanced projection of America’s growing racial and ethnic diversity (Davenport, 2020). Notably, the shifts move from broad racial categories toward more specific ones that better capture the immigrants’ racial and ethnic heritage.

Our estimates are, arguably, an understatement of the actual fluidity of racial and ethnic identities because our analytic approach is relatively conservative. We only recognize shifts into a particular racial and ethnic group when they are substantial enough to counter the natural decreases from mortality and emigration. Thus, the actual extent of race and ethnic fluidity is more pronounced than what is reported in this present study, making it an even more significant phenomenon to investigate further. However, our main substantive interest is not in the actual, precise magnitude of these shifts but how widespread (and how under-recognized and underappreciated in the scientific literature) the phenomenon has been. Hence, the relatively conservative approach adopted in this study is imperfect but adequate for capturing this often-overlooked phenomenon.

Future research shall attempt to gauge the exact magnitudes of the identity shifts within and across racial and ethnic groups among U.S. immigrants. The challenge, however, is that the members of each racial and ethnic group are a moving object and cannot be accurately and reliably traced and measured exactly because racial and ethnic identities are fluid. Thus, such attempts must require assumptions about the cohort-and-period-specific mortality and emigration rates for the original racial and ethnic groups. Considering that mortality fluctuates across groups and periods, those findings will be subject to discrepancies.

In conclusion, this study substantiates the fluid nature of racial and ethnic self-identification and the increasing prevalence of mixed-race identities among immigrants in the United States. The findings underscore the growing complexity and diversity within racial and ethnic categories, potentially challenging the traditional understanding of fixed identities. While our approach may understate the extent of this fluidity, it emphasizes the need for further research to accurately capture these dynamics. We expect future studies to refine the estimation of shifting identities and explore the implications for understanding the evolving racial and ethnic landscape of the United States.

## Data Availability

The datasets abstracted and analyzed for this study can be found in The Open Science Framework: https://osf.io/c52zy/view_only=e0924621a854437b8dfe6f2d872b499a.
