# Supplementary figures and images for: Changing self-identification among immigrants in the United States
Source: Front Sociol. 2025 May 9;10:1445287. doi: 10.3389/fsoc.2025.1445287 (PMC12098439; doi:10.3389/fsoc.2025.1445287)

**Appendix
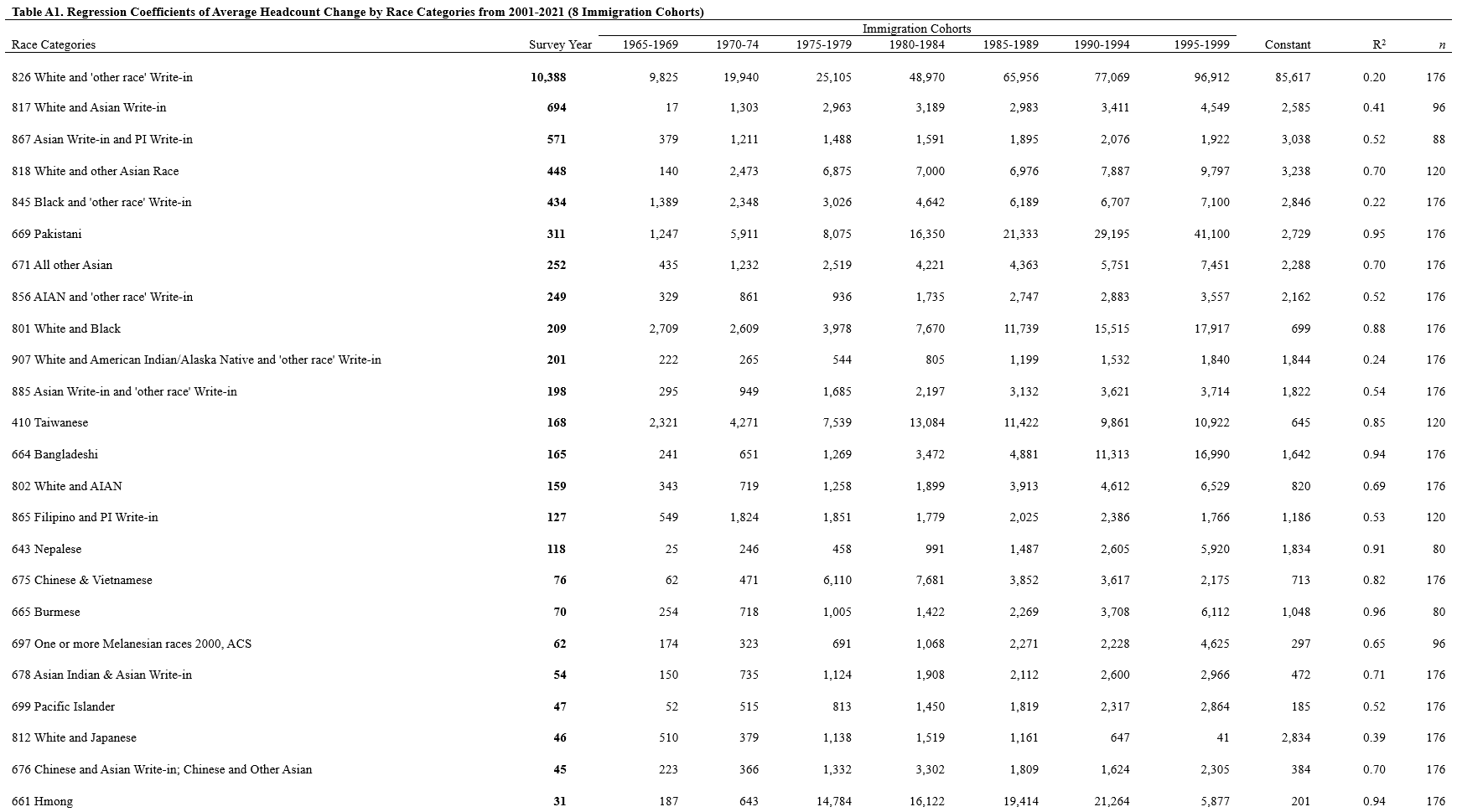

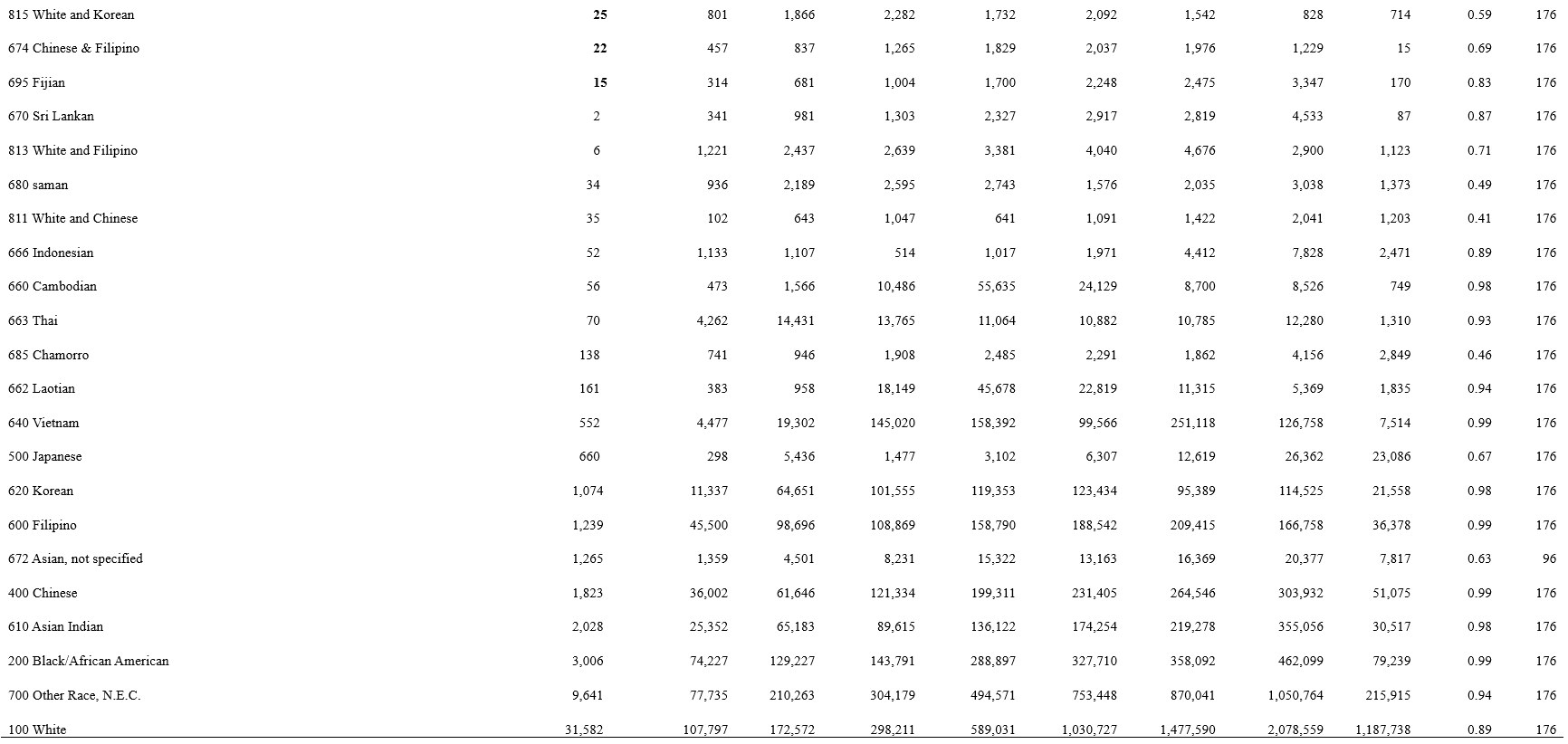
**

**
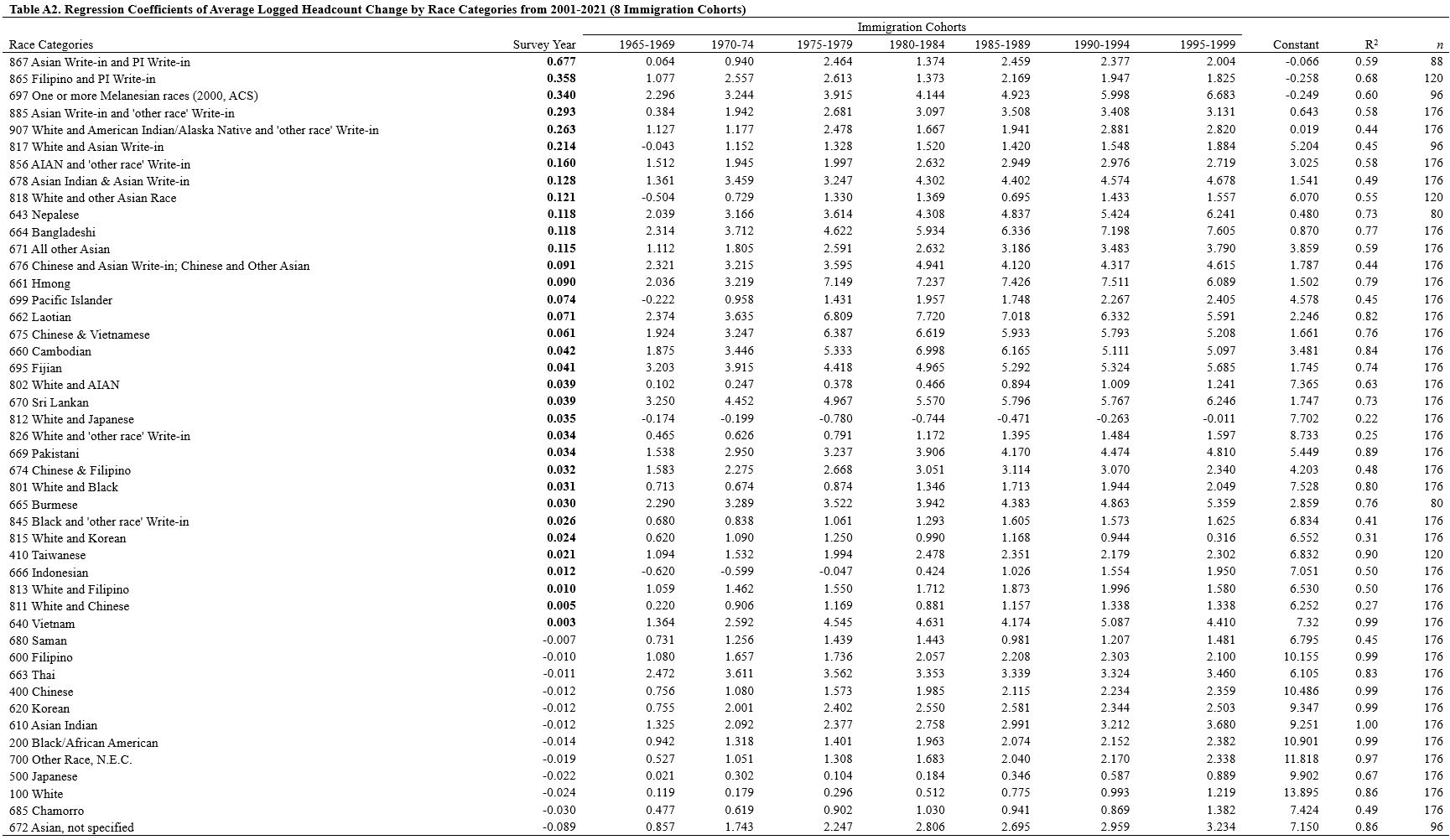
**

**
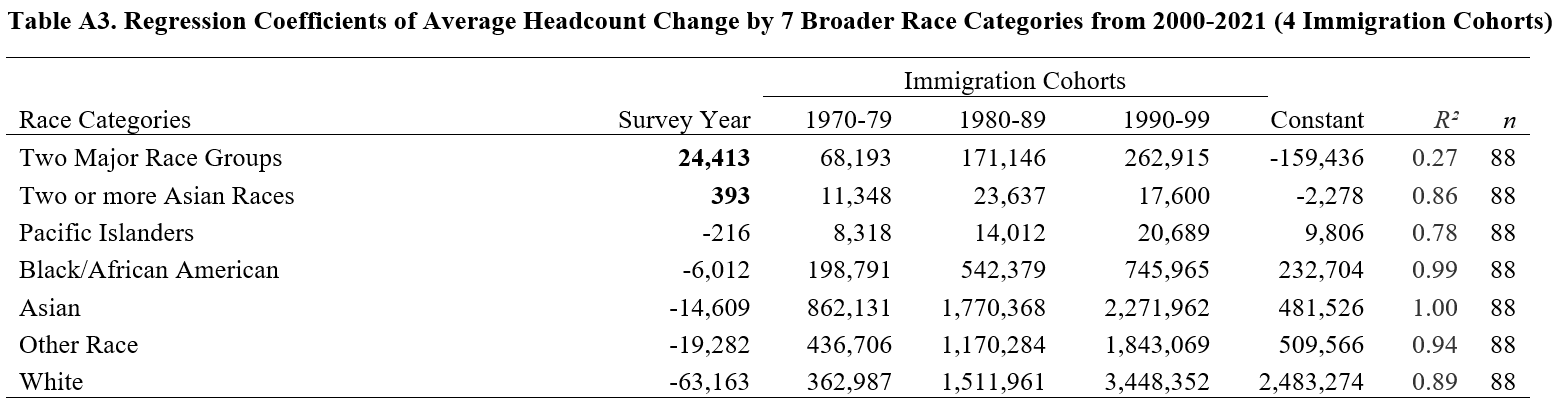

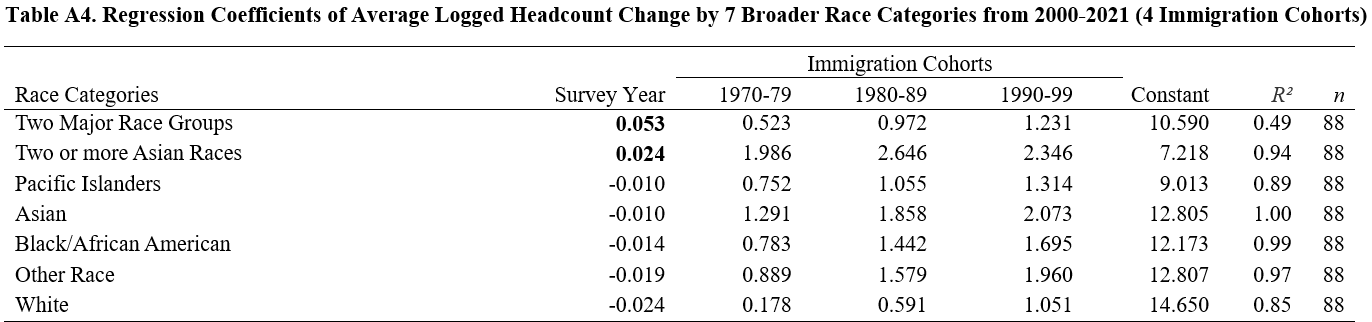
**

**
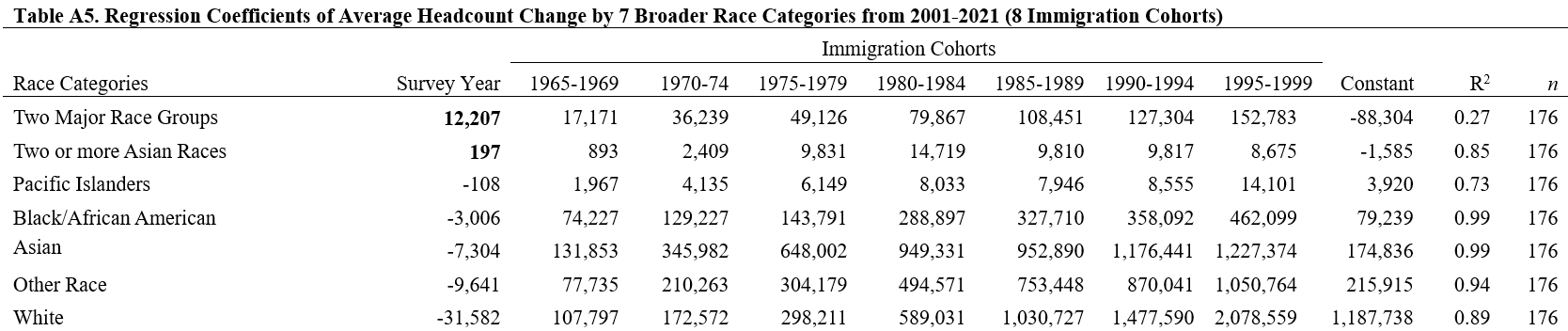

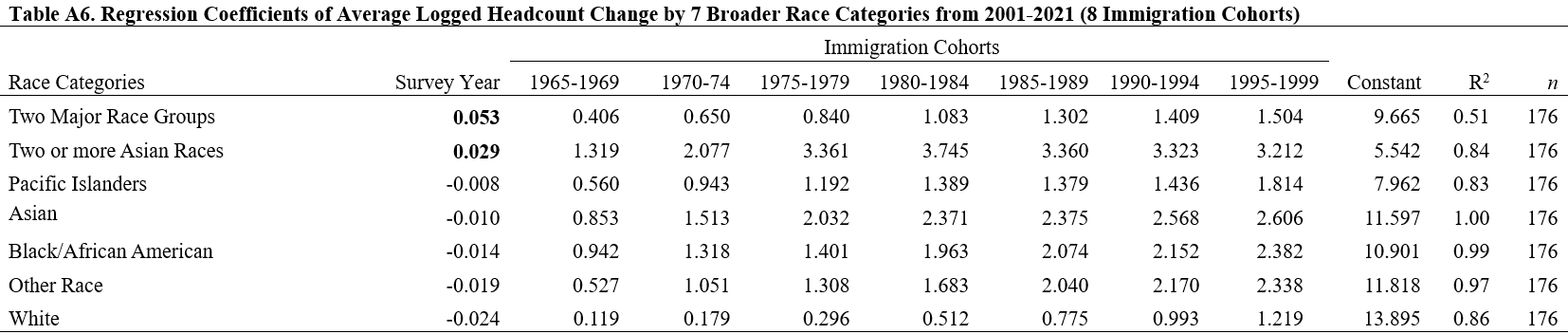
**

Supplement: Supplementary file 1 [file Table_1.docx]
